# Supplementary material for: Characterisation of an unusual nicotinic acetylcholine receptor subtype preferentially sensitive to biogenic amines
Source: Commun Biol. 2025 Dec 12;8:1764. doi: 10.1038/s42003-025-09143-z (PMC12700902; doi:10.1038/s42003-025-09143-z)
Supplement: Supplementary file 2 — Supplementary Material [file 42003_2025_9143_MOESM2_ESM.pdf]

Characterisation of an unusual nicotinic acetylcholine receptor subtype preferentially sensitive to biogenic amines

Eleanor L. Mitchell, Emily B. Armstrong, Franco Viscarra, Isabel Bermudez, Philip C. Biggin, James A. Goodchild and Andrew K. Jones

Supplementary Material

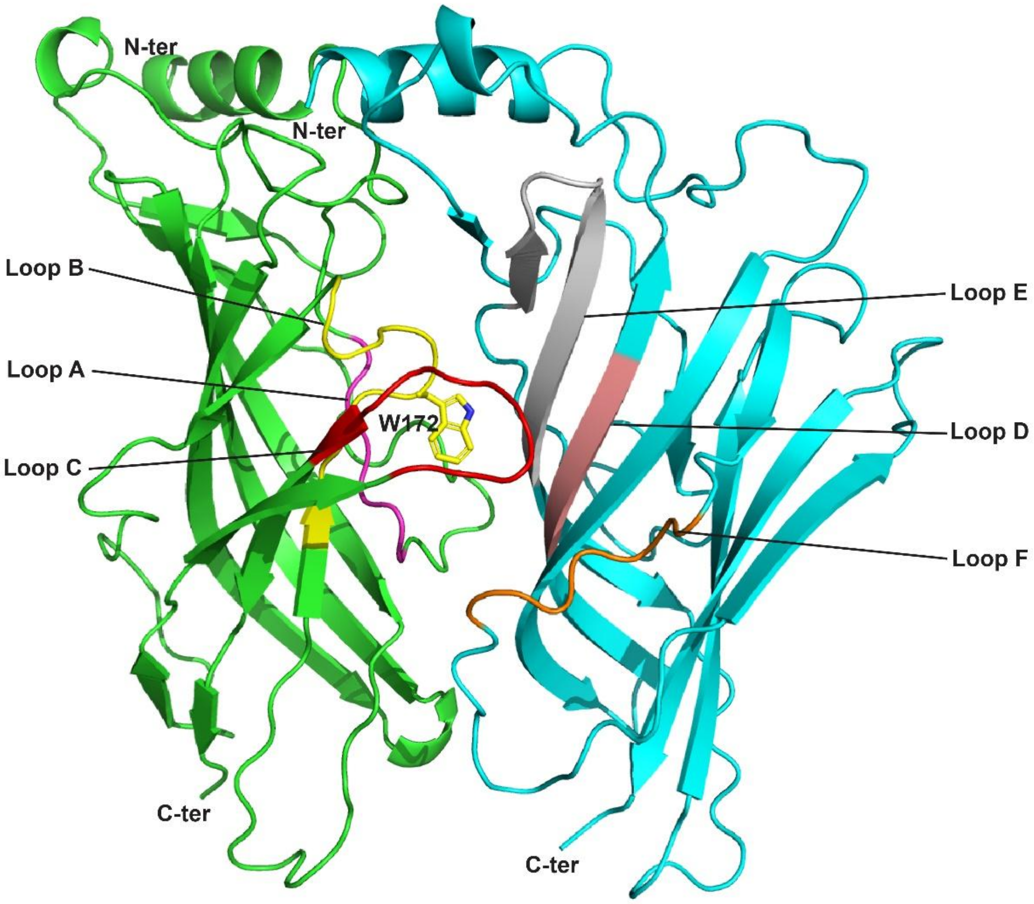

Supplementary Figure 1: Truncated portion of the *A. mellifera* α5 nAChR protein used for molecular dynamics simulations. Principal (+) and complementary (-) subunits are coloured green and cyan respectively. W172 is highlighted by sticks representation to showcase the position of the binding site with Loops A-F coloured in magenta, yellow, red, pink, grey, and orange, respectively.

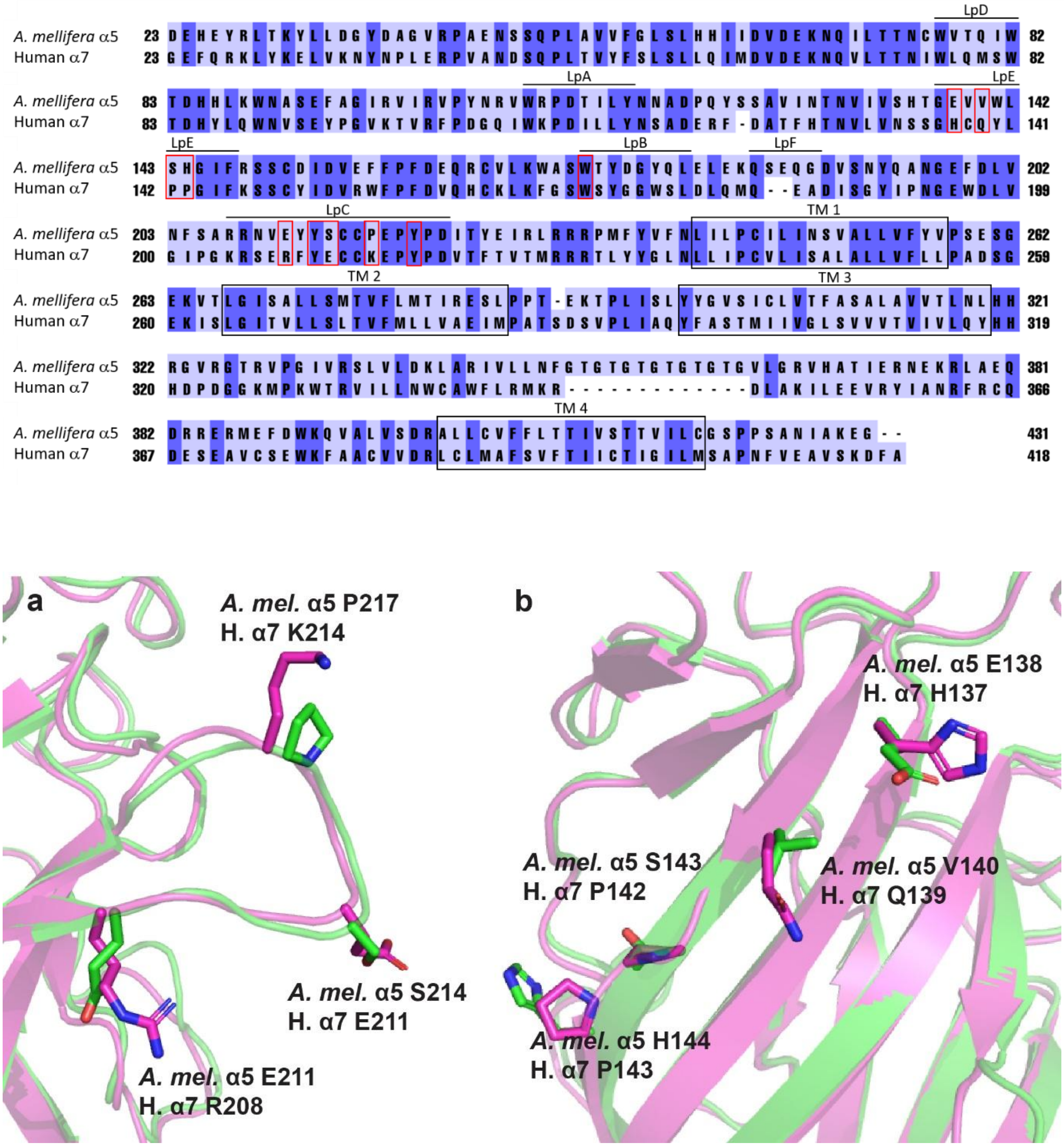

Supplementary Figure 2. Upper pane – Alignment of the *A. mellifera* α5 and human α7 nAChR subunit sequences used in homology modelling with the conserved amino acids highlighted in dark blue. The loops involved in ligand binding (LpA-F) as well as the four transmembrane domains (TM1-TM4) are indicated. Particular residues highlighted by three-dimensional modelling that were discussed in the text are shown in red boxes.

Lower panel – Superimposition of loop C (**a**) and loop E (**b**) between the *A. mellifera* α5 homology model (purple) and the human α7 nicotinic acetylcholine receptor (green). The residues that differ between the structures are highlighted in stick representation.

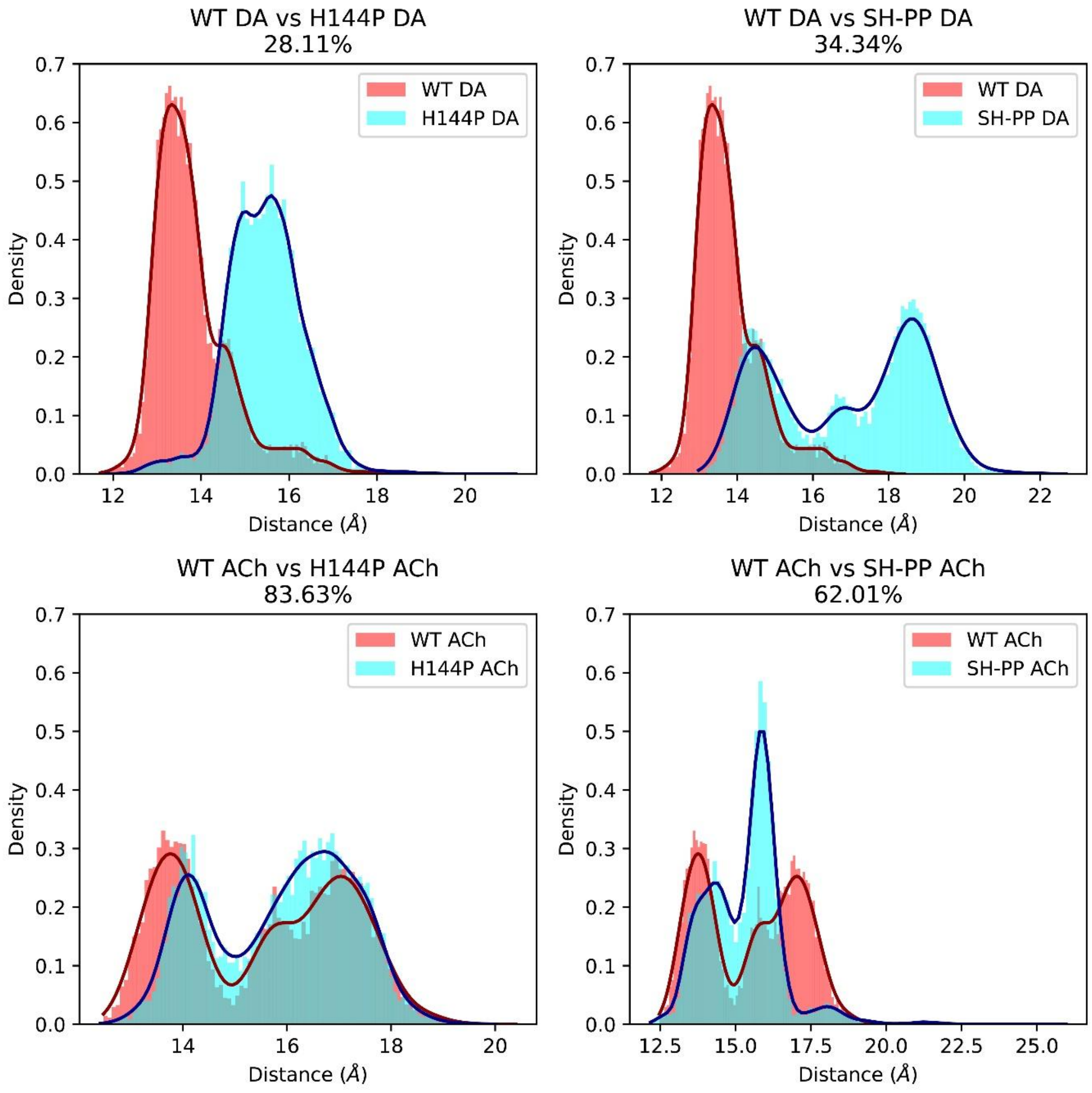

Supplementary Figure 3. Histogram distribution of the distance between the centre of geometry of loop C and the centre of geometry of the protein in wild-type vs mutant *A. mellifera* α5 nAChR proteins with dopamine (DA) or acetylcholine (ACh). SH-PP denotes the S143P+H144P double mutant. The dark outline shows the estimated probability density function used to calculate the overlap between distributions.

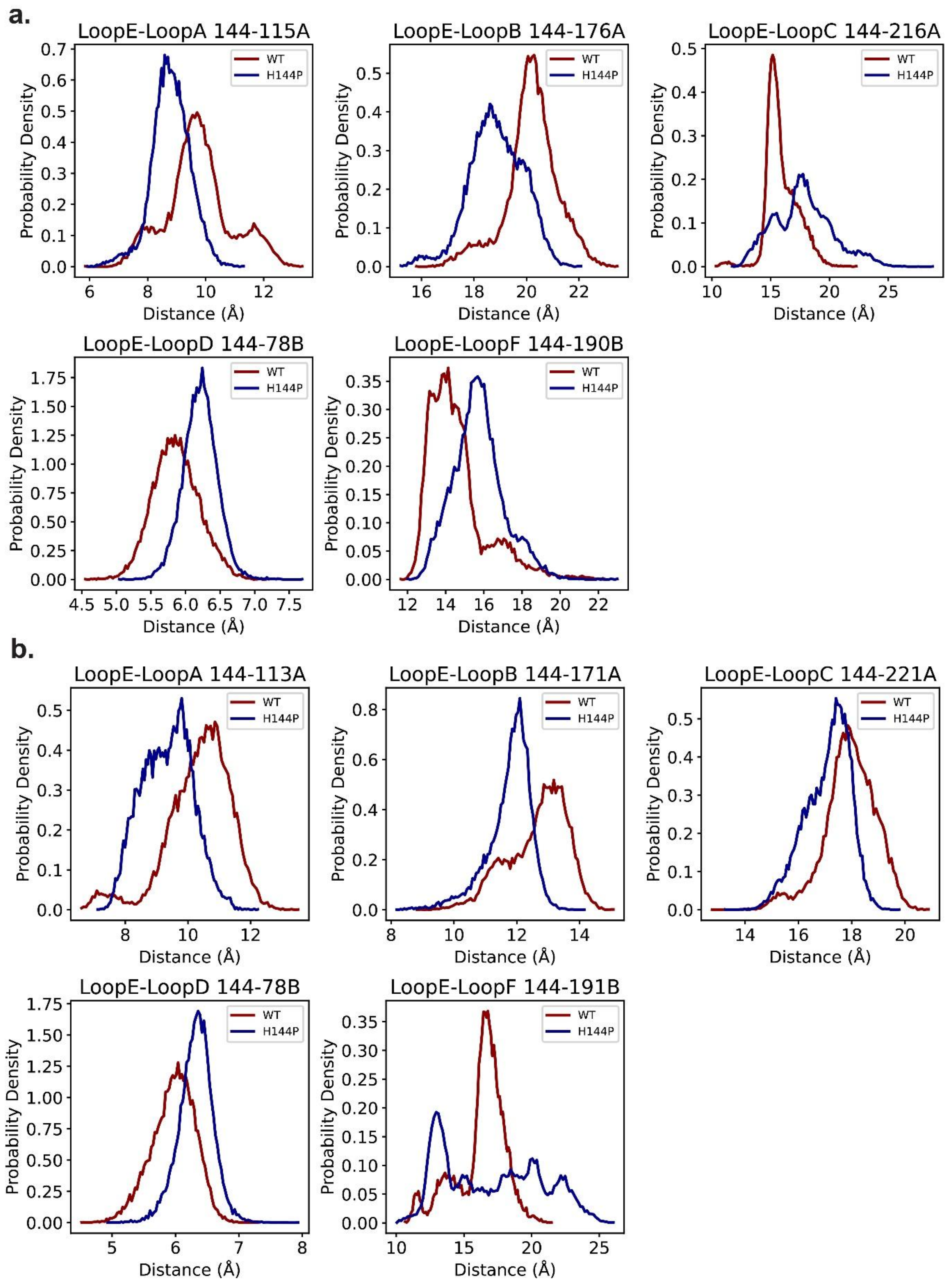

Supplementary Figure 4. Distribution of selected distances between residues of loops A, B, C, D and F with residue 144 of loop E for the wild-type vs mutant bound to dopamine (**a**) and acetylcholine (**b**).

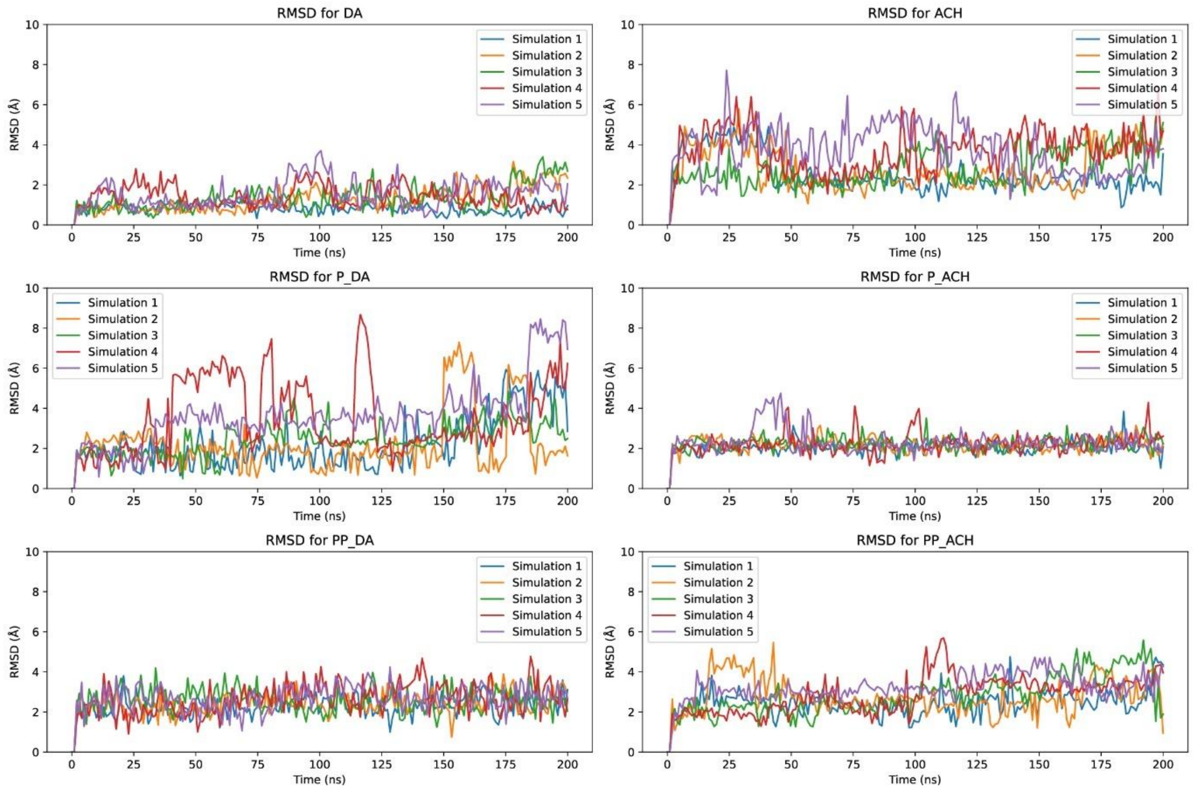

Supplementary Figure 5. RMSD (root mean square deviation) values of conventional MD simulations performed on each system starting from the centroid of each best cluster.

Supplementary Table 1. Primers used for site-directed mutagenesis of the *A. mellifera* α5 nAChR subunit. Amino acid residue numbering includes the N-terminal signal peptide.

Mutant	Primer	Sequence (5’ to 3’ direction)
E138H	Nucleotide 1 forward	GAGTCACACGGGGGACGTGGTGTGGTTGCC
	Nucleotide 1 reverse	GGCAACCACACCACGTCCCCCGTGTGACTC
	Nucleotide 2 forward	GAGTCACACGGGGCACGTGGTGTGGTTG
	Nucleotide 2 reverse	CAACCACACCACGTGCCCCGTGTGACTC
V139C	Forward	GTGAGTCACACGGGGCACTGTCAGTGGTTGCCCCCTGGG
	Reverse	CCCAGGGGGCAACCACTGACAGTGCCCCGTGTGACTCAC
V140Q	Forward	CACACGGGGCACGTGCAGTGGTTGCCCCCTGG
	Reverse	CCAGGGGGCAACCACTGCACGTGCCCCGTGTG
W141Y	Forward	GTCACACGGGGCACTGTCAGTATTTGCCCCCTGGGATATTTCGC
	Reverse	GCGAAATATCCCAGGGGGCAAATACTGACAGTGCCCCGTGTGAC
S143P	Nucleotide 1 forward	GGAGGTGGTGTGGTTGACCCCTGGGATATTTCGCAGCAGC
	Nucleotide 1 reverse	GCTGCTGCGAAATATCCCAGGGGTCAACCACACCACCTCC
	Nucleotide 2 forward	GTCACACGGGGCACTGTCAGTATTTGCCCCCTGGGATATTTCGC
	Nucleotide 2 reverse	GCGAAATATCCCAGGGGGCAAATACTGACAGTGCCCCGTGTGAC
H144P	Forward	GGAGGTGGTGTGGTTGAGCCCTGGGATATTTCGCAGCAGC
	Reverse	GCTGCTGCGAAATATCCCAGGGCTCAACCACACCACCTCC

Supplementary Table 2. Primers used in overlap extension PCR to replace loop C of *A. mellifera* α5 with the equivalent region from the *Homo sapiens* α7 nAChR subunit.

Stage	Primer	Sequence (5’ to 3’ direction)
1	5’ end*	ATCGCTCGAGATGTCGCCTTTGGTCCTGTTC
	Amela5Ha7lpC_Nterm	TTTGCAGCACTCATAGAACCTTTCACTCCTCTTCGCGGAGAAATTGACCAAGTC
2	Amela5Ha7lpC_Cterm	AAGAGGAGTGAAAGGTTCTATGAGTGCTGCAAAGAACCGTATCCTGACATCAC
	3’ end**	CGATCGTCTAGATTAACCCTCTTTGGCAATGTTCG
Overlap	5’ end*	ATCGCTCGAGATGTCGCCTTTGGTCCTGTTC
	3’ end**	CGATCGTCTAGATTAACCCTCTTTGGCAATGTTCG

* Underlined sequence corresponds to the *Xho*I restriction site, which was used to clone the subunit into the pCI vector (Promega).

** Underlined sequence corresponds to the *Xba*I restriction site, which was used to clone the subunit into the pCI vector (Promega).

Supplementary Table 3. Average exchange probabilities between replicas for each system.

System	Average Exchange Probability (%)
A. mel. α5 WT + DA	41.9 ± 4.7
A. mel. α5 + ACh	42.7 ± 4.9
A. mel. α5 H144P + DA	39.7 ± 4.7
A. mel. α5 H144P + ACh	41.7 ± 4.8
A. mel. α5 HS-PP + DA	48.9 ± 4.5
A. mel. α5 HS-PP + ACh	45.3 ± 4.7
