# Supplementary material for: Characterisation of an unusual nicotinic acetylcholine receptor subtype preferentially sensitive to biogenic amines
Source: Commun Biol. 2025 Dec 12;8:1764. doi: 10.1038/s42003-025-09143-z (PMC12700902; doi:10.1038/s42003-025-09143-z)
Supplement: Supplementary file 3 — Description of Additional Supplementary Files [file 42003_2025_9143_MOESM3_ESM.pdf]

Description of Additional Supplementary Files

File name: Supplementary Data 1
Description: Raw data producing graphs in Figures 1, 3, 4, 5 and 6.

File name: Supplementary Material - additional information and data supporting findings in the manuscript.
Description: Supplementary Material Figure 1: Truncated portion of the *A. mellifera* α5 nAChR protein used for molecular dynamics simulations.
Supplementary Material Figure 2. Upper pane – Alignment of the *A. mellifera* α5 and human α7 nAChR subunit sequences used in homology modelling with the conserved amino acids highlighted in dark blue. Lower panel – Superimposition of loop C and loop E between the *A. mellifera* α5 homology model and the human α7 nicotinic acetylcholine receptor.
Supplementary Material Figure 3. Histogram distribution of the distance between the centre of geometry of loop C and the centre of geometry of the protein in wild-type vs mutant *A. mellifera* α5 nAChR proteins with dopamine or acetylcholine.
Supplementary Material Figure 4. Distribution of selected distances between residues of loops A, B, C, D and F with residue 144 of loop E for the wild-type vs mutant bound to dopamine and acetylcholine.
Supplementary Material Figure 5. RMSD (root mean square deviation) values of conventional MD simulations performed on each system starting from the centroid of each best cluster.
Supplementary Material Table 1. Primers used for site-directed mutagenesis of the *A. mellifera* α5 nAChR subunit.
Supplementary Material Table 2. Primers used in overlap extension PCR to replace loop C of *A. mellifera* α5 with the equivalent region from the *Homo sapiens* α7 nAChR subunit.
Supplementary Material Table 3. Average exchange probabilities between replicas for wild-type and mutant *A. mellifera* α5 nAChR subunits.
